# A framework exploring the therapeutic alliance between elite athletes and physiotherapists: a qualitative study

**DOI:** 10.1186/s13102-021-00348-3

**Published:** 2021-10-09

**Authors:** W. M. Charmant, P. J. van der Wees, J. B. Staal, R. van Cingel, J. M. Sieben, R. A. de Bie

**Affiliations:** 1 MSc Human Movement Sciences – Physiotherapy, Maastricht University, Maastricht, The Netherlands; 2 Radboud Institute for Health Sciences, IQ Healthcare, Radboud University Medical Center, Nijmegen, The Netherlands; 3 Musculoskeletal Rehabilitation Research Group, HAN University of Applied Sciences, Nijmegen, The Netherlands; 4 Sport Medisch Centrum Papendal, Arnhem, The Netherlands; 5 Department of Anatomy and Embryology, Maastricht University and Caphri Research School, Maastricht, The Netherlands; 6 Department of Epidemiology and Caphri Research School, Maastricht University, Maastricht, The Netherlands; 7 Department of Acute Internal Medicine, Amsterdam UMC, VUmc, De Boelelaan 1117, 1081 HV Amsterdam, The Netherlands

**Keywords:** Elite athletes, Physiotherapists, Therapeutic alliance, Qualitative research

## Abstract

**Background:**

The therapeutic alliance (TA) is the bond between a patient and a physiotherapist during collaboration on recovery or training. Previous studies focused on the TA between physiotherapists and patients of the general population. Little information exists on whether this is similar in the demanding environment of elite athletes. The aim of this study was to investigate the components of TA between elite athletes and physiotherapists.

**Methods:**

Ten elite athletes and ten physiotherapists were interviewed using one-on-one semi-structured interviews between June 2020 and October 2020. Athletes were included if they competed at national or international championships. Physiotherapists had to treat elite athletes on a regular basis. Interview questions were based on TA components of the general physiotherapy population. The interviews were transcribed and coded using inductive thematic analysis.

**Results:**

The analysis resulted in an elite athlete TA framework which consists of nine themes and ten subthemes that could influence the TA. The nine themes are trust, communication, professional bond, social bond, elite athlete, physiotherapist, time, pressure and adversity, and external factors. This showed that the TA consists of bonds on different social levels, depends on the traits of both elite athletes and physiotherapists, and can be positively and negatively influenced by the external environment. The influences from the external environment seem to be more present in the elite athlete TA compared to the TA in the general physiotherapy setting. Multiple relations between themes were discovered. Trust is regarded as the main connecting theme.

**Conclusion:**

This study provides a framework to better understand the complex reality of the TA between elite athletes and physiotherapists. Compared to the general physiotherapy setting, new themes emerged. The created framework can help elite athletes and physiotherapists to reflect and improve their TA and subsequently improve treatment outcomes.

## Background

Elite athletes performing at the highest level of their respective sport can experience great achievements but also big deceptions. Musculoskeletal injuries are common and do not only cause physical pain but also psychological distress [1–4]. Recovery from these injuries may require the support of a physiotherapist who collaborates with the elite athlete towards a common goal. The success or failure of treatment depends on many factors. An important determinant of treatment outcome is the therapeutic alliance (TA), also referred to as helping alliance or working alliance [5]. Studies have investigated components of the TA in the general physiotherapy population. However, a gap of knowledge exists on the components of the TA in an elite athlete setting.

The TA, as established by Freud in 1912, is a sense of collaboration, warmth, and support between a patient and his or her therapist [6]. Later, in 1979, Bordin defined three main components which contributed to the TA: agreement on treatment goals, agreement on intervention, and an affective bond between patient and therapist [7].

Multiple studies have been carried out in psychological settings on the influence of the TA on treatment outcomes [8, 9]. In patients with musculoskeletal complaints, stronger TAs were associated with greater improvements in pain, function and disability [10, 11]. Moreover, a stronger TA leads to improvements in the ability to perform activities of daily living, specific physical tasks, global assessment of physical health and treatment satisfaction [5]. A partial explanation of this phenomenon could be the positive correlation between the TA and therapy adherence [5, 12].

No knowledge exists on whether the TA in an elite athlete setting is similar to the TA in a general physiotherapy population. The results from previous studies are based on populations treated for chronic low back pain, chronic musculoskeletal pain and brain injury among others [10, 11]. It is uncertain whether findings from the general physiotherapy population can be extended to the elite athlete setting. Other themes could be relevant in this professional sports environment in which physiotherapists are an important part of the team surrounding the athlete. Injuries are a frequent and impactful problem in elite athletes. This can cause long periods with high frequency treatments, increasing the presence of the physiotherapist in the athlete’s life. Elite athletes work through periods of performance and economic pressure, which cannot be compared to the general physiotherapy population. Moreover, severe injuries can be career ending for elite athletes. In elite sports, physiotherapists are not only present in times of injury but also play a role in return to play, prevention and the general physical well-being of their athletes. Therefore, the athletes and physiotherapists are more engaged with each other compared to the general physiotherapy population. Since the TA could have an impact on the treatment outcome, it is necessary to evaluate what the TA consists of between elite athletes and their physiotherapists [10, 11]. An increased understanding of the TA could improve care efficiency and improve treatment outcomes [1–3].

This qualitative study aimed to generate insight into what themes and factors the TA consists of between elite athletes and their physiotherapists. This knowledge can subsequently be used by elite athletes and physiotherapists to optimise their TA. It is hypothesized that the therapeutic alliance in the elite sports setting will include additional dimensions compared to the general physiotherapy population.

## Methods

### Research design

A qualitative study was performed to investigate the TA from the elite athletes’ and physiotherapists’ perspectives in the Netherlands. The Medical Ethical Committee of the academic hospital Maastricht and Maastricht University (azM/UM) approved this study (METC 2020-1521).

### Theoretical framework

Critical theory was used as research paradigm. The methodology for this study was grounded theory since the study was designed to discover what the social phenomenon ‘TA’ consists of according to elite athletes and physiotherapists [13, 14]. The ontology was ‘historical realism’ while the epistemology was ‘relative subjectivism’ [15].

### Participant recruitment and sampling

Participants were elite athletes and physiotherapists. Elite athletes were eligible if they were athletes competing at national or international championships and were registered a top sports status by the Dutch Olympic Committee*Dutch Sports Federation (NOC*NSF) [16]. To participate, the elite athletes must have been treated by a physiotherapist in the past six months. The elite athletes have their own personal physiotherapist and do not switch between physiotherapists. The elite athletes trained actively for 20 hours a week.

Physiotherapists were eligible to participate in this study if they treated elite athletes on a regular basis, at least once a week on average. Elite athletes and physiotherapists were excluded if they insufficiently mastered Dutch, defined as a level lower than 2F/B1.

Elite athletes and physiotherapists were recruited through Sports Medical Centre Papendal (SMCP). SMCP asked their physiotherapists to participate and subsequently asked their physiotherapists to recruit elite athletes for the study. Maximum variation sampling was used to include participants from multiple sports disciplines. Interested elite athletes and physiotherapists received written information about the project prior to the interview. Before starting the interviews, all participants were asked to sign the informed consent form.

### Setting

Interviews were held at SMCP to minimally disturb the participants’ schedules. SMCP is a sports medical centre for both elite athletes and the general population. SMCP is a part of Sport Centre Papendal which is a national high performance centre of the Dutch Olympic Committee for elite athletes where they can live, train and have access to multiple high-quality facilities. The interviews were performed between June 2020 and October 2020. Since the TA perceptions are personal and could differ per participant, interviews were held one-on-one to prevent interference in opinions by other participants. Each interview lasted 30–60 min. The interviews were performed by the first author (WMC).

### The general population TA framework

For the present study, the authors constructed a framework on current knowledge on TA in the general physiotherapy setting, hereafter referred to as the general population TA framework (“Appendix A”). This framework was used to derive questions for the semi-structured interview. The general population TA framework contains eight general themes mainly based on the review by Babatunde et al. and complemented by some other studies, all from the general physiotherapeutic setting [12, 17, 18]. These themes are: congruence, connectedness, communication, roles and responsibilities, expectation, influencing factors, individualised therapy and partnership [12].

However, no clear definitions of the themes could be derived from the work of Babatunde et al. [12]. Therefore, the following definitions were set for the themes in the present study. *Congruence* is defined as the agreement between elite athletes and physiotherapists on decisions during their collaboration. *Connectedness* is viewed as the social bond between both parties while *partnership* will be regarded as the professional bond. *Communication* concerns the process in which information is exchanged (non)verbally. *Expectation* is the belief that something will (not) happen in a certain scenario. *Individualized therapy* regards the physiotherapists’ patient-centred approach. The theme *roles and responsibilities* is defined as the physiotherapists’ professional attitude towards their elite athletes and vice versa. Finally, the *influencing factors* are external and personal characteristics influencing the TA.

### Interview guide

Interview questions were based on the general population TA framework. Follow-up questions were used to gain more insight in predefined and new themes. In addition, participants were asked to discard factors, from the general population TA framework, that they deemed not applicable in a TA between elite athletes and physiotherapists (Table 1). After the first few interviews, an interim analysis was performed to improve the interview guide.

**Table 1:** Interview guide

Topic	Elaboration/example questions
Introduction	Introduction by the interviewer on the purpose of the interview, including creation of a safe atmosphere. TA will be introduced and explained
General TA	What do you think as elite athlete/physiotherapist that is important for a strong TA?
Theme 1: congruence	What is your view on the importance of agreement between you and your elite athlete/physiotherapist and what influences the agreement process?
Theme 2: connectedness	What do you regard as important for the connection between you and the elite athlete/physiotherapist in a social context?
Theme 3: communication	Which factors do you think contribute to a proper communication between a physiotherapist and an elite athlete?
Theme 4: expectation	How do you think that your expectation/the elite athlete’s expectation affects the TA? What could this expectation be based upon?
Theme 5: individualised therapy	Do you think an individualised therapy contributes to a stronger TA? What makes you believe that the therapy is based on you/the elite athlete?
Theme 6: influencing factors	What do you think contributes to a strong TA as an external factor? What elite athlete’s or physiotherapist’s personal characteristics, do you think contribute to a strong TA?
Theme 7: partnership	What do you view as important for the professional bond between you and the elite athlete/physiotherapist?
Theme 8: Roles and responsibilities	What do you see as the elite athletes’ or physiotherapists’ professional roles, what do these consist of?
New themes	What do you as elite athlete/physiotherapist see as other important factors regarding the TA? How do you feel that the TA in an elite athlete setting differs from the general population TA?
Discarding previous factors	Participants received a printed version of “Appendix A”. They were asked to remove factors which they felt did not contribute to the TA in an elite athlete environment

### Data collection

After signing informed consent, participants completed a questionnaire regarding their demographic characteristics. For the elite athletes this consisted of age, sex, sport discipline, years of performing at elite athlete level and average number of physiotherapist visits in a week. Physiotherapists were asked to fill out a questionnaire concerning their age, sex, specialization and years of experience in physiotherapy in general and years of physiotherapy experience in elite sports.

The interviews were recorded and transcribed verbatim. Permission for recording the interview was part of the informed consent. Recordings and transcriptions received a code to which only the researchers had access and were saved on a secure server at Maastricht University. After transcription, the recordings were deleted. All data (questionnaires, interviews and quotes) were processed in coded form and not retraceable to individual elite athletes and physiotherapists.

### Data analysis

The author listened to the audio recordings and read the transcripts to familiarize himself with the data. An inductive thematic analysis was performed, starting with initial coding in which important (groups of) words were labelled using Nvivo [19]. During subsequent intermediate coding, categories were subsumed under other categories [19]. During advanced coding, categories were combined into overarching themes [19]. All themes were evaluated to determine whether they influenced the TA. From these themes, a bottom-up framework of the elite athlete TA was constructed. Inductive thematic saturation, which relates to the emergence of new codes or themes, was achieved after analysing ten elite athlete and ten physiotherapist interviews. The minimum sample size limit for grounded theory, 20 interviews as defined by Creswell, was achieved [20]. The framework was sent to the participants for a member check. Furthermore, a meeting with two additional elite athletes and two additional physiotherapists was held for validation of the framework. During the data analysis process, multiple meetings with all authors occurred for their input on the analysis.

### Quality criteria for qualitative research

To optimize credibility, researchers from Maastricht University, Radboud University, HAN University and SMCP collaborated in this study [21]. Furthermore, a member check was performed with all participants who agreed to participate [21]. To improve transferability, elite athletes and physiotherapists specialised in a number of different sports were interviewed to make the results applicable to multiple sport disciplines [21]. Moreover, a detailed description of the participants is provided (Table 2) [21]. By iterative data collection and data analysis, a consistent and systematic method approach was used to collect all possible variability within the results to ensure dependability [21]. In order to provide insight into possible bias of the researcher, reflexivity was pursued to increase confirmability [21–23].

**Table 2:** Demographic characteristics of elite athletes (n = 10) and physiotherapists (n = 10)

Characteristics	N1	N2	N3	N4	N5
Profession	EA	EA	EA	EA	EA
Age	20–24	20–24	15–19	25–29	25–29
Sport	400 m hurdles	BMX	Volleyball	Judo	Badminton
Status	HP	Talent	A	A	A
Years active as EA	1	4	3	8	14
Average PT visits/week	1–2	2	5	2	2

	N6	N7	N8	N9	N10
Profession	EA	EA	EA	EA	EA
Age	15–19	20–24	15–19	20–24	15–19
Sport	Table tennis	Heptathlon	Handball	60 m/100 m sprint	Volleyball
Status	Talent	A	Talent	A	Talent
Years active as EA	4	1	2	4	2
Average PT visits/week	0	2	2	2	3

	N11	N12	N13	N14	N15
Profession	PT	PT	PT	PT	PT
Age	35–39	35–39	30–34	30–34	40–44
Sport	Judo	Handball	Table tennis	Badminton	BMX
	Volleyball				Motocross
	Water polo				
Specialisation	Manual therapist and movement scientist	Sport and manual therapist	Manual therapist	Sport therapist	Manual therapist
Years active as PT	9	13	4	11	14
Years active as PT in elite sports	6	13	3	11	8
Average EA visits/week	15	34	2	10	4–5

	N16	N17	N18	N19	N20
Profession	PT	PT	PT	PT	PT
Age	25–29	40–44	30–34	25–29	30–34
Sport	Volleyball	Road cycling	Road cycling	Volleyball	Athletics
	Badminton	Badminton			
Specialisation	None	Sport and manual therapist	Movement scientist and PhD	Sport therapist	Manual therapist
Years active as PT	3	21	13	8	10
Years active as PT in elite sports	2	12	10	2	8
Average EA visits/week	8	9	20	20–25	40

EA = Elite athlete, PT = Physiotherapist, HP = high potential status

#### Reflexivity

At the time of the study, the researcher WMC was a 22-year old male MSc Human Movement Sciences student, specialisation Physiotherapy at Maastricht University. Prior to the master, he graduated at SOMT University of Physiotherapy for his BSc of Physiotherapy in 2019. WMC is an active football player himself, though not on a professional level. Furthermore, he closely follows multiple professional sports disciplines.

As part of the reflexivity, peer debriefing was performed with a peer, L.V., who was not involved in the study. This debriefing evaluated whether themes remained true to the data and determined whether the themes were accurately represented during the analysis-synthesis process. Multiple meetings between the authors were held to discuss the themes, factors and the framework. The final framework was also discussed with two elite athletes and two physiotherapists who did not participate in the interviews.

## Results

**Fig. 1:**
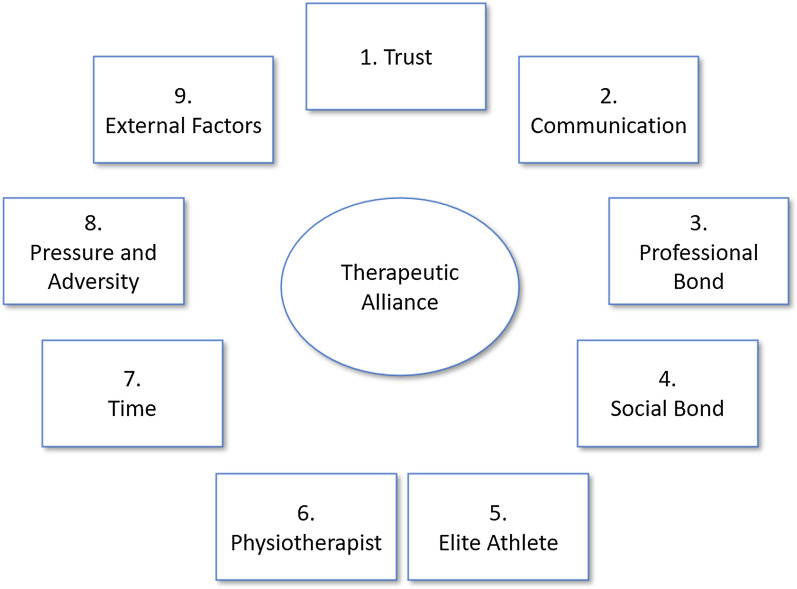
A visualisation of the nine themes in the elite athlete TA framework

**Fig. 2:**
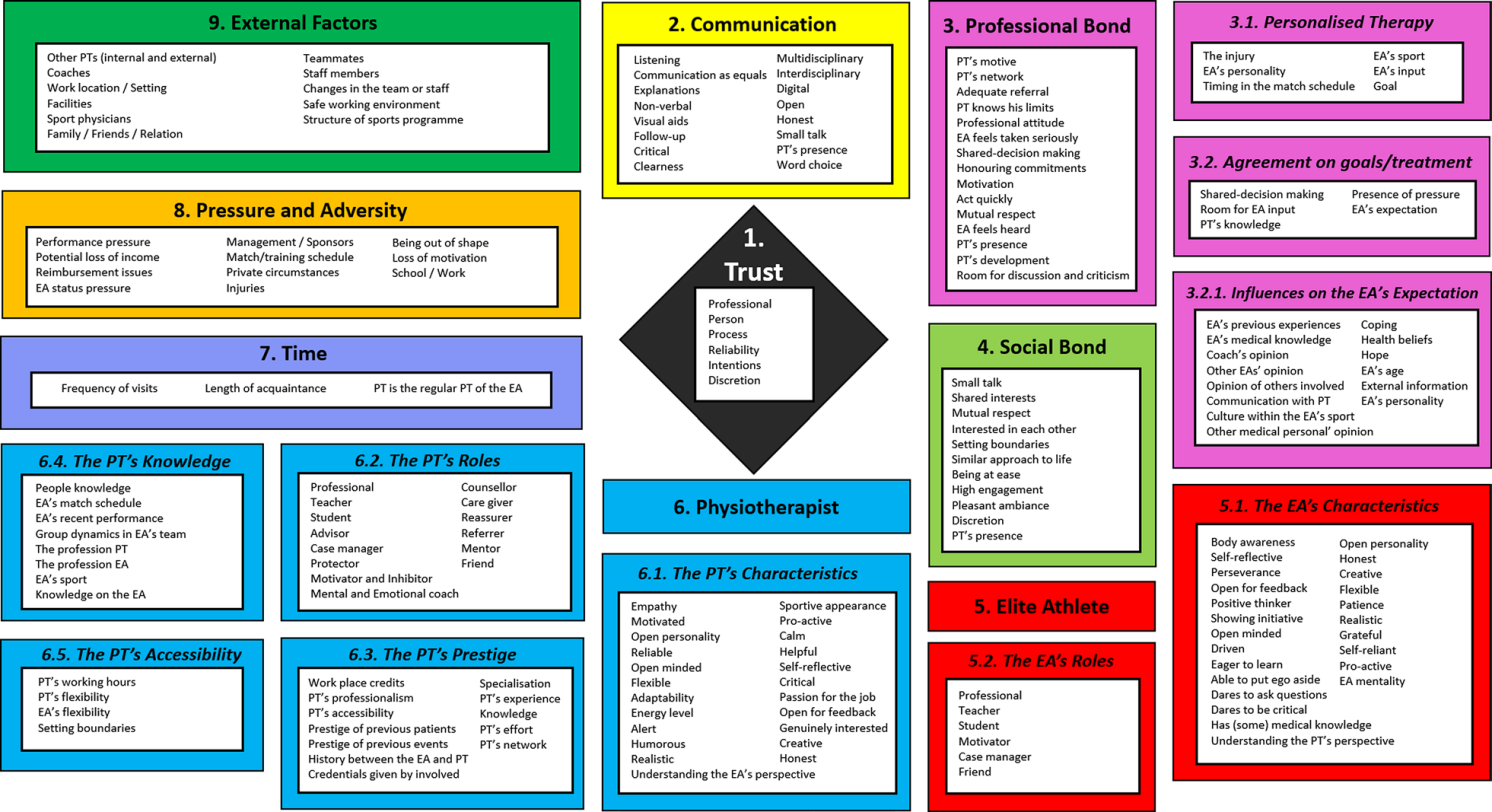
The elite athlete—therapeutic alliance framework. PT = Physiotherapist, EA = elite athlete

Twenty interviews were conducted with ten elite athletes and ten physiotherapists (Table 2). Two more elite athletes and two more physiotherapists participated in a discussion on the final framework (Table 3). In total nine men and 15 women participated. The data from the interviews was analysed and nine themes, ten subthemes and 189 factors were distinguished (Figs. 1 and 2).

**Table 3:** Demographic characteristics of the elite athletes (n = 2) and physiotherapists (n = 2) participating in the final framework discussion

Characteristics	N21	N22
Profession	EA	EA
Age	25–29	20–24
Sport	Archery	Handball
Status	A	Talent
Years active as EA	10	4
Average PT visits/week	1	4

Characteristics	N23	N24
Profession	PT	PT
Age	40–44	30–34
Sport	Skiing and snowboarding	Archery and shooting
Specialisation	Manual therapist	Manual therapist
Years active as PT	19	13
Years active as PT in elite sports	13	10
Average EA visits/week	5	2

In this article the themes, subthemes and highlight factors of the themes are elaborated. A complete overview of all 189 factors within the elite athlete TA Framework is presented in Fig. 2. The presented results are not prerequisites for a strong TA since the demand for certain aspects will differ per TA.

### Trust

Trust between elite athletes and physiotherapists is one of the most important themes related to TA as it was mentioned after the first open question by 16 out of the 20 participants (Table 1):

According to me, it [the TA] is entirely dependent on trust (PT-A).

Trust is characterised by trust in the professionality of the physiotherapists and elite athletes. Furthermore, trust in each other as a person and trust in the therapeutic process were stated as important. The elite athletes want to trust the physiotherapists that they will do everything for their elite athletes with the best intentions. Finally, there has to be trust between the elite athletes and physiotherapists that private conversations will remain private.

### Communication

According to the participants, both parties need to see each other as equals and should listen carefully to one another. Moreover, communication could have a critical note to improve the personal and therapeutic process:

I think the why-question can be the most critical question because someone can write a whole exercise for you but if you ask why should I do it, you [the physiotherapist] must be able to explain. …. I think it [being critical] is very important because eventually it is your [the elite athlete’s] career. Afterwards, you don’t want to say but he [the physiotherapist] said that I should do it so I just did that. You always have to be critical, also in this area [physiotherapy] (EA-A).

In order to increase communication, elite athletes would like to see their physiotherapists present during events, such as competitions or training. However, communication should not only be about the elite athlete’s injuries or sports. Physiotherapists explained that small talk can also be a tool to discuss difficult subjects. Elite athletes expressed that small talk is very important for good communication and for social bonding. Contrary, the absence of small talk can affect the social bond:

With one physiotherapist you can start quite a conversation, while another physiotherapist, who is quiet and doesn’t talk, only talks about your injury. Personally, I would rather have a conversation about, I don’t know, boys with my physiotherapist instead of only talking about my injury, because that doesn’t really cheer me up (EA-B).

Apart from communication with elite athletes, interdisciplinary and multidisciplinary communication are important components in the TA. A lack of good interdisciplinary communication with other physiotherapists or multidisciplinary communication will negatively affect the TA. Multidisciplinary communication is considered as the communication between physiotherapists and coaches, sport physicians, surgeons, dieticians, psychologists, strength trainers, assistant coaches and life-style coaches.

### Professional bond

Concerning the professional bond, participants stated that there should always be mutual respect:

When you notice that someone [the physiotherapist] is willing to go through fire for you, that creates mutual respect and from my side even more goodwill to do everything that they [the physiotherapists] would tell me to do (EA-C).

Helping the elite athletes should be the main motive of the physiotherapists and not for their own gain in e.g. reputation. Moreover, elite athletes should feel taken seriously and listened to. Elite athletes should be involved in shared-decision making. There should be room for discussion and criticism to improve the process. Elite athletes appreciate it when their physiotherapists keep continuously developing themselves since this correlates with the elite athlete’s continuous process of development.

#### Personalised therapy

As part of the professional bond, a personalised treatment plan will result in increased appreciation and subsequent motivation:

When you receive a tailor-made treatment plan, you know that someone has put a lot of time and energy into it which results in trust. If I would receive a regular treatment plan which I felt was taken from the internet or made in a hurry, I wouldn’t be motivated (EA-C).

#### Agreement on goals/treatment plan

Agreement on goals or treatment plans are essential components of a successful collaboration within the professional bond. To achieve agreement, it is important that there is room for an elite athlete’s input to actively involve the elite athlete. Moreover, achieving agreement can be influenced by the presence of pressure, the physiotherapist’s knowledge and the elite athlete’s expectations.

##### Influences on the elite athlete’s expectations

These expectations are largely influenced by personal factors and opinions of others. The culture within the elite athlete’s sport can play a role in their expectations as some physiotherapists highlighted the differences in coping between different sports. External information such as posts on social media by famous athletes about their recovery can define the elite athlete’s expectations.

### Social bond

The social bond is about similarities and a trustworthy environment. Small talk will help increase the social bond and make elite athletes feel at ease. Elite athletes will be able to tell their physiotherapists more, which requires discretion on behalf of their physiotherapists concerning the obtained information. This is one of the factors that elite athletes and physiotherapists find most important:

*What I find important is that they [the elite athletes] can share their private situations in confidence which can go from anorexia to serious home sickness to suicidal thoughts (PT-C).*

The intensity of the social bond is influenced by the boundaries set by both elite athletes and physiotherapists regarding availability. The more physiotherapists are available for their elite athletes, the better the social bond will be:

She [the physiotherapist] was with me at the Olympic Games and there I really liked it that she was in the stands. You just knew that she was there (EA-C).

### Elite athlete

The theme about the elite athlete regards the elite athlete’s characteristics and the elite athlete’s roles within the TA. The characteristics are generally characteristics of the elite athlete related to independence, taking responsibility for their own recovery and motivation. The elite athlete’s roles can help the elite athlete but also the physiotherapist in achieving a stronger TA.

#### The elite athlete’s characteristics

Elite athletes should have the ability of self-reflection and be open for feedback in order to improve themselves and the therapeutic process. The elite athletes who think positive and realistic will be more capable of dealing with their recovery. Furthermore, elite athletes who dare to ask questions and be critical will be able to improve the therapeutic process. By understanding the perspective of the physiotherapist, elite athletes will achieve a stronger TA. Elite athletes should be able to put their own egos aside in order to accept certain adversities.

#### The elite athlete’s roles

Next to characteristics, elite athletes can portray certain roles within the TA. The most obvious one being the role of a professional who takes his/her job seriously and wants to do whatever it takes in order to achieve progress. Sometimes in order to learn from their physiotherapists, elite athletes have to become students who are willing to learn. On the other hand, elite athletes can also be teachers when for example explaining sport-specific information to their physiotherapist. Elite athletes can also play the role of motivators for their physiotherapists and some elite athletes regard themselves as friends of the physiotherapist. Elite athletes can portray the role of case manager:

I think the elite athlete is a case manager as well. It is elite athlete’s body and the elite athlete’s career, so being assertive and taking initiative is important. I think it is very important that the elite athlete takes responsibility for his career and does not put it in the hands of anyone else (PT-D).

### Physiotherapist

The theme physiotherapist consists of five subthemes; physiotherapist’s characteristics, roles, prestige, knowledge and accessibility.

#### The physiotherapist’s characteristics

It is very important that physiotherapists are adaptive in their relation with their elite athletes:

Since there are many differences between athletes, it requires another approach per athlete and situation. So you [the physiotherapist] should have a certain degree of adaptability or chameleon like abilities. You need to know how and when you should or shouldn’t trigger someone (PT-B).

Physiotherapists should also possess the ability of self-reflection and be critical towards the process. They should also be alert for changes or other relevant signals in the elite athlete’s life. Furthermore, the physiotherapists should have personal traits that aim at actively helping the elite athlete while being open for improvement.

#### The physiotherapist’s roles

Apart from being the professional, physiotherapists could also be teachers when they educate their elite athletes or the elite athletes’ family or friends. However, physiotherapists should also be students, since both physiotherapists and elite athletes indicate that it would be beneficial if physiotherapists would be open to learning from their elite athletes. As advisors and mentors, physiotherapists can guide their elite athletes. Sometimes, physiotherapists will be confidants or counsellors to whom elite athletes can talk about subjects that they can’t discuss with others:

Look, some elite athletes share a lot of information which is beyond the physiotherapeutic domain. You [the physiotherapist] are sort of a psychologist, so when someone has an issue which is not related to the injury than it will increase the alliance between therapist and athlete (PT-D).

During times of adversity, physiotherapists can become mental and emotional supporters for their elite athletes. They will also act as motivators or inhibitors depending on their elite athlete’s character. Another role showing that physiotherapists are more than just physiotherapists, is the care giver role. During tournaments or training camps, physiotherapists will often take on other side jobs like preparing food or materials for their elite athletes in order for elite athletes to fully focus on their training.

One of the more important roles is that of protector of the elite athlete’s mental and physical health. One of the participants told a story in which a coach and a physiotherapist did not agree on the severity of an elite athlete’s injury. The coach was convinced that the injury was less severe than the physiotherapist indicated. The elite athlete continued training with the coach which resulted in a serious aggravation of the injury. This could have been prevented if the physiotherapist had stood up for his elite athlete. The elite athlete later heard that the physiotherapist knew that this could have happened which eventually led to the end of their collaboration.

#### The physiotherapist’s prestige

The physiotherapist’s specialisation, e.g. manual therapist or sport therapist, provides prestige since it shows additional knowledge. The credits of the work place can also play a role in prestige:

You go to a physiotherapist of whom you know that he really has a lot of knowledge, otherwise he wouldn’t work here at Papendal (EA-D).

Physiotherapists will have more prestige if they have already treated a world class athlete within the discipline of that particular elite athlete or if they have been to prestigious events such as the Olympic Games. The physiotherapist can also gain prestige due to credentials given by coaches, teammates, other physiotherapists, staff members or others involved.

#### The physiotherapist’s knowledge

A beneficial effect on the TA will be observed if the physiotherapist expands his knowledge by learning information on the sport, the match schedule and recent performances of their elite athletes. Moreover, knowledge regarding the profession elite athlete, people knowledge in general and knowledge on the specific elite athlete will be beneficial:

I think that is important because if a [position A] has an inner thigh or hamstring injury than she wouldn’t be able to play but a [position B] would be able to play. Therefore, I think it is important that you know which position someone plays and what is important for that position (EA-B).

Finally, knowledge regarding group dynamics within the elite athlete’s team can be helpful.

#### The physiotherapist’s accessibility

The importance of the physiotherapist’s accessibility or availability is illustrated by this quote:

Within elite level sports, I find it important to be available 24/7 for an elite athlete since they do everything for it and it is all about the small differences that can make the difference between winning, losing and achieving goals (PT-D).

Not all physiotherapists agree with a 24/7 mentality but all emphasise that they agree on certain rules with their elite athletes in order to be accessible as demonstrated in the following quote:

What I agreed upon with my elite athletes is that if it is serious you can call me 24/7 but for every minor thing you know where to find my room or you can text me. Then I’ll estimate how serious it is (PT-E).

### Time

A small, yet important theme, is time. In general this theme states that the more positive encounters the elite athlete and physiotherapist have, the stronger their TA can become. The longer elite athletes and physiotherapists know each other, the stronger the TA can be.

### Pressure and adversity

The theme pressure and adversity explains multiple situations that can create stress on the TA. These situations can be related to the elite athlete’s sport, financial or personal situations. The elite athlete can feel pressure to perform at a certain level due to qualification criteria or financial consequences due to e.g. forced absence.

Besides pressure, elite athletes can experience adversity when they are out of shape, suffering from an injury or losing motivation. The number of therapeutic visits during these times will increase in which the TA can get stronger or weaker. During this time, it becomes more important for physiotherapists to show empathy, show involvement and be accessible. Physiotherapists can be someone for elite athletes to talk to about the adversity and receive mental support.

### External factors

The last theme is the external factors which mainly consists of people or environmental factors that can influence the TA. Changes in a team’s roster or staff members can affect the TA. For example, if an elite athlete changes coaches, the new coach may communicate with the physiotherapist differently compared to the old coach. As a result, the TA between a physiotherapist and their elite athlete can change.

The structure of the sports programme is a factor that can influence the physiotherapist’s authority regarding the elite athlete’s healthcare. Some programmes are coach-controlled meaning that the coach will make the final decision for anything regarding the elite athlete and the physiotherapist can only provide an advice. This can heavily influence the position of the physiotherapist within the team surrounding the elite athlete and can have consequences for the health of the elite athlete. Finally, a safe working environment in which physiotherapists feel that their decisions are supported by the rest of the team or staff, is important. This was illustrated by the following quote:

Can you assume that the rest of the team [or staff] is behind you or is it more the snake pit that top sport can be in which everyone starts pointing fingers. I know both situations. One in which a team is there as a team and has trust in each other. The other time it is more like she said this and I would have never done that. That does not feel safe for a physiotherapist (PT-F).

## Discussion

The objective of this study was to discover what the TA between elite athletes and physiotherapists consists of. Nine main themes with subsequent subthemes and factors were discovered using semi-structured interviews with ten elite athletes and ten physiotherapists. These results emphasized the importance of trust, communication, connection and effort of both parties for a strong TA. Trust can be regarded as the foundation of the TA as it is related to all other themes. The investigation on TA in this specific setting showed new results compared to what was known on the TA in the general population physiotherapy setting. There are no studies on the effect of TA in EA. We believe that the positive correlation from the psychological therapy and the general physiotherapy settings will be applicable in the elite sports setting [8–11].

### Comparing the elite athlete setting with the general population setting

When comparing the TA between an elite athlete setting and the TA in the general population, multiple differences are present. The treatment goals differ between elite athletes and the general population. While people from general population might want to return to work, hobbies or sport, elite athletes want to return to and excel in their sport due to the high-performance standards and competitive culture in elite sports.

The environment surrounding elite athletes is different compared to regular patients due to the presence of coaches, national sports associations and other stakeholders. These can interfere and also provide additional pressure in the TA. However, one of the positive effects of the environment surrounding elite athletes is the accessibilities of certain facilities, e.g., elite athletes will receive medical imaging tests within 24 h if necessary. Furthermore, the physiotherapists state that they have to act quicker when treating elite athletes compared to regular patients. Finally, elite athletes and physiotherapists see each other more often compared to physiotherapists and regular patients. This results in a more personal bond and higher levels of trust.

### Comparing the elite athlete TA framework to the general population TA framework

Comparing the final elite athlete TA framework to the general population TA framework shows some of these differences and more (Fig. 2 and “Appendix A”) [12, 17, 18]. The theme partnership has been changed into the professional bond in which more emphasis is laid on input from both the elite athlete and physiotherapist, helping the elite athlete being the main motive for the physiotherapist and the physiotherapist’s capabilities [12, 18]. Congruence, known as agreement on goals/treatment plan in the elite athlete TA framework, has been submerged under the professional bond while the expectation has been submerged under agreement on goals/treatment [12]. Moreover, individualised therapy was deemed a component of the professional bond named personalised therapy [12]. Connectedness has been turned into the social bond which does not show personal characteristics since those were included in the themes elite athlete and physiotherapist [12]. The characteristics, roles and responsibilities have all been included in the subthemes of elite athlete and physiotherapist [12]. Therefore, showing the characteristics and roles separately for elite athletes and physiotherapists as opposed to the combined presentation in the general population TA framework [12]. Some additional subthemes, such as prestige, knowledge and accessibility, have been added in the physiotherapist theme.

The themes time and, pressure and adversity, are mentioned in the elite athlete TA framework but not in the general population TA framework. It is very likely that time and pressure will also play a role in the TA of the general population. However, pressure is less likely to be present in a similar degree in the general population TA compared to the elite athlete TA.

One of the biggest problems both elite athletes and physiotherapists experienced with the general population TA framework was the term prerequisites. They stated that one could not make characteristics like humour and life-experience a requisite for a strong TA. Instead of a prerequisite, it would become a characteristic that could be beneficial but is not required.

### Relations between themes

The nine themes have multiple overlapping areas and show multidirectional relationships between them. This is, for example, present in the relations between the theme ‘trust’ and all other eight themes. The communication can strengthen or weaken trust, e.g. when communication is not clear resulting in doubt. The stronger the professional or social bond, the stronger the level of trust can be due to achieved successes or a strong personal connection. The characteristics of both elite athletes and physiotherapists can determine whether they will trust each other. The amount of time elite athletes and physiotherapists know each other can influence the level of trust, e.g., an elite athlete and a physiotherapist who have just met each other will probably have a lower level of trust compared to an elite athlete and physiotherapist after a five-year collaboration. Pressure or adversity may cause stress and lower levels of trust due to doubt or uncertainties. Finally, some external factors such as coaches or others involved may cause an increase or decrease in trust levels between an elite athlete and physiotherapist due to their opinion about the physiotherapist. This shows the complex reality of the TA in a professional sports setting in which multiple themes can affect one another.

### Strengths and limitations

The sample size is scientifically acceptable for the purpose of this study. Inductive thematic saturation was achieved as no new concepts emerged in the last interviews. The minimum amount of interviews for grounded theory studies, 20 interviews, was achieved [20].

Elite athletes of different sports and with different status participated in this study providing different views from different cultures regarding the TA. Together the physiotherapists were active in eleven different sports which also provided experience from different perspectives and their respective cultures. A member check was performed to enhance credibility, in which 19 participants agreed with the final elite athlete TA framework. One participant wasn’t available for the member check due to pregnancy leave.

A peer debriefing regarding the themes was performed. Moreover, multiple panel discussions with all authors took place to discuss the results. Members of the panel had experience in the field of elite sports and research. A meeting with two additional elite athletes and two additional physiotherapists was held to validate the framework.

As part of the reflexivity, WMC’s interpretation of the results could have been biased towards a more physiotherapist point of view due to his degree in physiotherapy. To prevent this as much as possible, coded items in Nvivo were not separated based on profession of the participant. Therefore, all parts of all interviews that could have been quoted for a certain topic were shown simultaneously, with the researcher not knowing to whom these quotes belonged. As such, while constructing the final elite athlete TA framework, the researcher could not bias towards a certain point of view as he could not deduce the role of the quoted interviewee.

### Transferability of the results

The results of the present study were derived from a sample of elite athletes and physiotherapists active in the Netherlands in multiple sport disciplines and in different stages of their careers. The authors believe that the results are, potentially, applicable to elite sport settings all over the world. However, the specific Dutch setting does not warrant easy extrapolation for elite athletes and their physiotherapists in the rest of the world. The external environment and the culture might differ due to different sport programme structures or different hierarchical relations between those involved. Nonetheless, the TA will mainly concern the elite athlete and physiotherapist in which their connection is the most important. Thus, implying the importance of the themes in the TA.

## Conclusion

This qualitative research showed the themes, subthemes and factors related to the TA in elite athletes and physiotherapists, of which trust was a main factor. Furthermore, it showed the relations between the themes and provided insight into what could influence the themes. New themes such as time, and pressure and adversity, have been discovered in the new elite athlete TA framework compared to the general population TA framework. The final result of this research is an elaborated framework which can aid both elite athletes and physiotherapists in reflecting on their part of their own TA. By creating a stronger TA with their elite athletes, physiotherapists can achieve better treatment outcomes and contribute to the performance of the elite athlete. Moreover, physiotherapy educational institutions and elite sports organisations can use this information to increase their knowledge on the TA.

## Recommendations

The authors recommend that professional sports medical centres with physiotherapists distribute the final framework among their physiotherapists and elite athletes to stimulate self-reflection and subsequently improve the TAs. Interventions might be used to improve TAs when necessary using the insights provided by the elite athlete TA framework.

Further research can be conducted to investigate the current gap of knowledge regarding the factors that influence the relation between physiotherapists and the elite athletes’ coaches. This can provide relevant insight due to the importance of the coach in the TA.

Another area in which knowledge is lacking is whether the TA between Paralympic elite athletes and their physiotherapists is significantly different compared to the elite athlete TA framework.

## Data Availability

The datasets generated during and/or analysed during the current study are not publicly available due to the signed agreement with the participants that the transcribed interviews would only be available to the authors.

## Appendix A: The general population TA Framework

The general population TA framework consists of eight themes according to Babatunde et al. 2017 [12]. Each theme is further elaborated into factors. Factors in bold were regarded as most commonly described within a theme in the Babatunde et al. study. * = the factors’being present’ and’being receptive’ have been added to the theme connectedness and the factor’commitment’ has been added to the theme’partnership’ based on research of Miciak [18], ** = The factor’transparency’ is added to the theme’communication’ based on results of Crom [17].

## Abbreviations

EA: Elite athlete
PT: Physiotherapist
TA: Therapeutic alliance

## References

[1] Saragiotto BT, Di Pierro C, Lopes AD (2014). Risk factors and injury prevention in elite athletes: a descriptive study of the opinions of physical therapists, doctors and trainers. Braz J Phys Ther.

[2] Junge A, Engebretsen L, Mountjoy ML, Alonso JM, Renström PA, Aubry MJ (2009). Sports injuries during the summer Olympic games 2008. Am J Sports Med.

[3] von Rosen P, Heijne A, Frohm A, Fridén C, Kottorp A (2018). High injury burden in elite adolescent athletes: a 52-week prospective study. J Athl Train.

[4] Shuer ML, Dietrich MS (1997). Psychological effects of chronic injury in elite athletes. West J Med.

[5] Hall AM, Ferreira PH, Maher CG, Latimer J, Ferreira ML (2010). The influence of the therapist-patient relationship on treatment outcome in physical rehabilitation: a systematic review. Phys Ther.

[6] Freud S. The dynamics of transference. In: Classics in psychoanalytic techniques. 1912;3–8.

[7] Bordin ES (1979). The generalizability of the psychoanalytic concept of the working alliance. Psychother Theory Res Pract.

[8] Shattock L, Berry K, Degnan A, Edge D (2018). Therapeutic alliance in psychological therapy for people with schizophrenia and related psychoses: a systematic review. Clin Psychol Psychother.

[9] Kallergis G (2019). The contribution of the relationship between therapist-patient and the context of the professional relationship. Psychiatrike.

[10] Ferreira PH, Ferreira ML, Maher CG, Refshauge KM, Latimer J, Adams RD (2013). The therapeutic alliance between clinicians and patients predicts outcome in chronic low back pain. Phys Ther.

[11] Kinney M, Seider J, Beaty AF, Coughlin K, Dyal M, Clewley D. The impact of therapeutic alliance in physical therapy for chronic musculoskeletal pain: a systematic review of the literature. Physiother Theory Pract. 2018;1–13. 10.1080/09593985.2018.1516015 30265840

[12] Babatunde F, MacDermid J, MacIntyre N (2017). Characteristics of therapeutic alliance in musculoskeletal physiotherapy and occupational therapy practice: a scoping review of the literature. BMC Health Serv Res.

[13] Teherani A, Martimianakis T, Stenfors-Hayes T, Wadhwa A, Varpio L (2015). Choosing a qualitative research approach. J Grad Med Educ.

[14] Korstjens I, Moser A (2017). Series: practical guidance to qualitative research. Part 2: context, research questions and designs. Eur J Gener Pract.

[15] Bergman E, de Feijter J, Frambach J, Godefrooij M, Slootweg I, Stalmeijer R (2012). AM last page: a guide to research paradigms relevant to medical education. Acad Med.

[16] NOC*NSF. TeamNL Topsportstatus. Available from: https://nocnsf.nl/athlete-services/teamnl-topsportstatus.

[17] Crom A, Paap D, Wijma A, Dijkstra PU, Pool G (2020). Between the lines: a qualitative phenomenological analysis of the therapeutic alliance in pediatric physical therapy. Phys Occup Ther Pediatr.

[18] Miciak M, Mayan M, Brown C, Joyce AS, Gross DP (2018). The necessary conditions of engagement for the therapeutic relationship in physiotherapy: an interpretive description study. Arch Physiother.

[19] Chun Tie Y, Birks M, Francis K (2019). Grounded theory research: a design framework for novice researchers. SAGE Open Med.

[20] Creswell JW, Poth CN (2016). Qualitative inquiry and research design: choosing among five approaches.

[21] Korstjens I, Moser A (2018). Series: Practical guidance to qualitative research. Part 4: trustworthiness and publishing. Eur J Gener Pract.

[22] Austin Z, Sutton J (2014). Qualitative research: getting started. Can J Hosp Pharm.

[23] Dodgson JE (2019). Reflexivity in qualitative research. J Hum Lact.

